# An energy-gradient theory of embedded predictions in the brain

**DOI:** 10.3934/Neuroscience.2026016

**Published:** 2026-06-23

**Authors:** Kuzma Strelnikov

**Affiliations:** 1 Centre for Cognitive and Brain Sciences, University of Macau, Taipa, Macau SAR, China; 2 Department of Public Health and Medicinal Administration, Faculty of Health Sciences, University of Macau, Macao SAR, China

**Keywords:** gradients, predictions, energy, fMRI, spatial coding

## Abstract

Activity gradients measured with neuroimaging play a fundamental role in brain function, yet their relationship to the brain's internal predictive models during rest remains poorly understood. Clarifying this relationship can reveal how the brain processes information efficiently and adapts to a changing environment. Here, I discussed how energy flows may give rise to gradients of activity between voxels and to spatial coding in fMRI, and I described computational algorithms that can be applied directly to brain-activity images. I proposed that the brain continuously maintains and updates predictions about its environment through internal models, and that these models are embedded in the dynamic activity flows observed at rest and during cognitive tasks. This perspective emphasizes the role of energy turnover in supporting cognition: Neural circuits reflect predictions and adjustments shaped by past experience. I argue that the interplay between predictive processing and energy dynamics offers a richer account of cognitive mechanisms and points to new research directions in psychology and physiology. Together, these insights underscore the importance of energetic principles in brain physiology.

## 1. Internal models

The central idea behind brain prediction is that the brain holds internal models of the environment. Before entering an office, for example, we already hold a model of a typical office: It has a floor, a ceiling, furniture, tables, chairs, and windows. A cage holding a monkey in that office would violate this model. The mismatch produces surprise [1] and demands further analysis: Why is the monkey there, and how should we respond? Resolving such unexpected situations inevitably increases glucose consumption and energy turnover in the brain.

Perceptual expertise enables us to recognize familiar objects as wholes (“this is an office”) without analyzing every detail, so that a known object or situation can be identified quickly [2]. When the input matches an internal model, the corresponding concept is activated rapidly; we think “this is a bird” upon seeing a typical bird. A creature lacking wings or a beak, however, does not match our model of a bird. We then analyze its parts and attempt to integrate them into a whole, even when the resulting image matches no model [3].

Recognizing an object as a whole means holistic perception, supported by diffuse projections from the thalamus to the cortex. Integrating details into a whole, by contrast, is sequential processing that is hierarchical and organized across levels [4],[5]. The cortex is arranged accordingly: Lower-level regions process basic sensory features, and higher-level regions integrate this information to generate predictions [6]. During stimulation, lower-level regions respond to basic inputs, while higher-level regions form more general predictions about the expected input. Even basic input, however, is compared with basic internal representations, such as the edge and contrast detectors of the visual cortex [7].

The internal representations present in the resting brain therefore provide the basis for predicting ordinary situations [3]. In predictive coding, the brain continually predicts the incoming sensory information and compares these predictions with the actual input. A mismatch generates a prediction-error signal that propagates through the neural hierarchy [8]. During stimulation, such error signals appear as changes in neural activity relative to the resting state, particularly in regions that generate predictions.

When similar information arrives repeatedly, the brain forms corresponding neural circuits that persist in the resting state as new internal models. These models are continuously updated as new sensory information arrives, and neural responses across cortical regions change accordingly. Predictive coding also proposes that the brain weighs predictions and prediction errors according to their estimated reliability or precision. Neural responses may therefore reflect precision-weighted prediction errors, with stronger responses signaling more reliable and informative errors [7],[8].

Predictive coding involves top-down and bottom-up interactions between cortical regions. Top-down signals carry predictions from higher-level regions, whereas bottom-up signals convey sensory information and prediction errors from lower-level regions. During stimulation, the interaction between these signals is visible in the dynamics of neural activity across the cortical hierarchy.

The visual cortex illustrates this process. The primary visual cortex (V1) processes basic features such as oriented edges and contrast [9]. When a visual stimulus reaches the eye, V1 neurons respond to specific features in the input and generate bottom-up sensory signals [10]. Higher-level areas, such as the secondary visual cortex (V2) and the ventral visual stream, receive these signals and construct predictive models of the visual scene, learning to associate particular patterns of V1 activity with particular objects and configurations [6].

Based on these models, higher-level areas generate top-down predictions of the expected input and send them to lower-level areas such as V1 to anticipate incoming information. The bottom-up signals from V1 are compared with these top-down predictions, and any mismatch generates a prediction-error signal [3].

The error signals then propagate up the hierarchy, informing higher-level areas that their models require updating [7]. These areas use the errors to refine their models and learn to make more accurate predictions. As the brain encounters further sensory information, this process of model adaptation continues, improving predictive performance over time [8].

Internal representations are never exact; they are probabilistic. The brain generates predictions and maintains probability distributions over possible sensory causes, so that perception can be understood as approximate inference within an internal generative model, analogous to Bayesian inference in statistics. Formally, the brain performs this inference by minimizing variational free energy [1],[11], which provides an upper bound on surprise. By continuously updating its probabilistic representations and enabling neural dynamics to settle on the best-fitting explanation, the brain keeps surprise low. More surprising information requires greater cognitive effort and metabolic energy to resolve.

This account shows how predictive coding explains the interaction between lower-level sensory processing and higher-level predictive modeling in the visual cortex. Similar principles apply to other sensory modalities and cognitive processes [11]. Mapping predictive-coding mechanisms onto observed patterns of neural activity can clarify how the brain efficiently processes and interprets the sensory world [12].

## 2. Brain predictions and specialized networks

**Figure 1.**
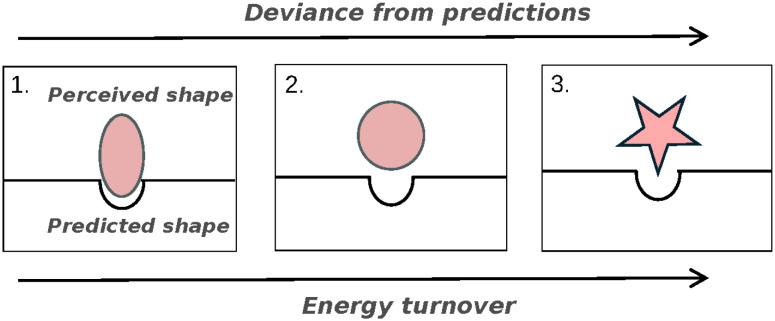
Energy demands and the precision of predictions. Prediction precision and energy demand during stimulation are inversely related. Increased precision (e.g., in state 1) reduces deviance detection and energy needs. This enhanced precision, likely facilitated by glutamatergic mechanisms such as long-term potentiation, optimizes synaptic processing of expected stimuli and reduces overall energy consumption. Conversely, lower precision (e.g., state 3) requires functional reorganization within neural populations, increasing energy demands by recruiting additional circuits and excitation loci.

From the discussion above, the brain's main function can be understood as the detection of changes in the environment. When the environment matches the brain's expectations, it requires no further analysis. In the resting state, without stimulation, the brain already contains expectations and models of the environment; the electrical activity and energy turnover observed at rest therefore reflect the maintenance of these internal models. When information arrives, it is compared with the expectations derived from the models (Figure 1).

Such a comparison requires additional activity and energy. If the incoming information matches expectations, little further analysis is needed and changes from the resting state are minimal. If it differs markedly from expectations, detailed and attentive analysis is required, increasing electrical activity and energy turnover. The expectations that form internal models are built from information received repeatedly during sensory experience. For example, we have shown that resting brain activity differs between people with normal hearing and those with cochlear implants, and that this difference becomes more pronounced as experience with the implant increases [13]. In other words, memory traces of frequent sensory stimulation form the basis of internal models.

How does this explain the specialized activity of brain networks? Different regions store different memory traces and environmental representations, and the properties of these representations correspond to the known specializations of particular cortical areas: The auditory cortex represents sound frequencies, and the visual cortex represents shapes. The underlying mechanisms may differ, with candidates including neural adaptation for short-term memory and synaptic plasticity for long-term memory. The crucial step is the comparison of stored traces with incoming information: Input is analyzed against the most similar representations across the cortex, minimizing the energy required. When stimulation is progressively degraded, energy minimization in specialized areas becomes less efficient, and brain activity shifts accordingly [14].

At the neural level, one possible account of memory traces is that the brain compares changes in synaptic parameters with the ranges those parameters have occupied over a given period. These ranges are stored in memory and are formed by repeated neural activity consolidated through feedback mechanisms. Memory traces of repetitive stimulation can thus serve as the basis for prediction. Long-term potentiation (LTP), mediated by N-methyl-D-aspartate (NMDA) receptors, is a leading candidate for the physiological basis of memory traces [15]. LTP occurs in many regions, including the hippocampus, cortex, amygdala, and cerebellum, and strengthens synaptic transmission for several hours or days. After repetitive stimulation, the brain generates spontaneous activity that predicts a stable environmental state, facilitating the perception of expected changes through LTP. LTP arises when presynaptic depolarization is synchronized with postsynaptic depolarization, a learning rule grounded in previous experience. I have previously proposed the following stages for processing expected and unexpected information through LTP [16]:

1. Sensory receptors register the physicochemical parameters of the environment.

2. Sensory information reaches the brain, where it alters the pattern of spontaneously generated activity.

3. This spontaneous activity predicts the next likely state of the environment; after repeated input, neural predictions of environmental conditions are formed.

4. The perception of expected environmental changes by the appropriate neural subsystem is enhanced at the synaptic level by mechanisms such as LTP.

5. If the incoming information matches the prediction, processing follows the path pre-formed by LTP. The response is typical, no new adaptive profile is created, and energy is saved because the neural circuits need not be adapted to the input.

If the incoming information does not match the prediction, processing leaves the LTP-facilitated path. Free energy increases, silent synapses are switched on, additional circuits form, and new excitation loci appear.

## 3. Neuroenergetic basis

Energy is a fundamental concept that helps explain how entities, from objects to living organisms, interact and influence one another. It provides a common language for connecting very different phenomena without requiring every underlying detail. The energy contained in the food we eat, for instance, is converted into the electrical activity that powers brain cells; we can describe this link in terms of energy without specifying every biochemical step. At the most basic level, energy relates to motion and position: A moving object possesses kinetic energy, and an object positioned so that it could begin moving possesses potential energy. A ball resting on a table, for example, has potential energy because it could roll off and start moving.

Energy is a fundamental property of physical systems and continually shifts between forms. In an isolated system, even at the molecular and cellular scale, the total internal energy is the sum of all kinetic and potential energies present, and this internal energy can power processes such as the movement of molecules in brain cells. Most real systems, however, including the body and brain, are not perfectly isolated, and some internal energy inevitably escapes to the environment as heat. Only a portion of the internal energy is therefore available for useful work, and the more organized and efficient the system, the more of its internal energy it can convert into useful outcomes. The portion available for work is called free energy (“free” meaning available to do work).

The brain can be described as a complex molecular system. It receives energy as glucose molecules and thermal energy from the bloodstream, which raises the kinetic and potential energies of its molecular components and thus its overall internal energy. Part of this internal energy powers the brain's metabolic processes and functions, while the remainder is dissipated as heat. Free energy is of particular interest because it represents the fraction of internal energy that can be directed toward useful work; the portion available to drive the brain's activities and operations. In summary, the brain receives energy inputs, converts them into increased internal energy, and uses the free-energy component to perform its necessary functions and processes.

The brain's primary energy source is glucose from the bloodstream. Glucose is a molecule of six carbon atoms, and the bonds between these atoms store potential energy that can power brain activity. Releasing this energy involves two major stages. The first is the anaerobic stage, which does not require oxygen: In the cytoplasm, the six-carbon glucose molecule is split into two three-carbon pyruvate molecules. This stage is relatively inefficient, producing only a small amount of adenosine triphosphate (ATP), the cell's energy carrier. The second is the aerobic stage, which requires oxygen and occurs in the mitochondria, where the two pyruvate molecules are broken down, and the carbon atoms bind to oxygen. This stage is far more efficient, generating up to 38 ATP molecules per glucose molecule.

In both stages, the energy released from breaking the carbon–carbon bonds is captured in the high-energy bonds of ATP. ATP is then transported throughout the cell to power biochemical processes and to raise the kinetic and potential energies of brain molecules. Oxygen is critical to this process: Without it, the anaerobic stage alone yields only about four ATP molecules per glucose molecule, rather than the 38 possible with full aerobic metabolism.

The ATP produced from glucose is used primarily to establish electrochemical gradients across cell membranes [17]. In other words, most of the energy from glucose is converted into the potential energy of electromagnetic fields stored across the membrane. This potential energy is then transformed into the kinetic energy of ions, which move across the membrane and create energy flows that propagate in various directions through the brain. These flows are transformations of energy that travel along cellular structures such as axons, dendrites, and synapses [18]. Because local changes in metabolic energy turnover propagate slowly, over seconds. they are transmitted instead as faster electromagnetic-field changes that occur within milliseconds.

Thus, the energy from glucose is converted into the energy of electrochemical gradients and then into the energy of ion movement. Such energy flows along and between cellular structures enable the rapid transmission of information within the brain.

## 4. Activation as a reorganization of brain activity

No exact definition of “activation” exists. Intuitively, the term refers to a brain region being more active than at some less demanding functional level. Increased activity may mean a higher neuronal firing rate, greater regional cerebral blood flow, or increased oxygen and/or glucose consumption, depending on the energetic demands of the task. Despite these varied and often indirect measures, brain activation is best understood as a degree of energy transformation within an ensemble of neuroglial units, which different methods capture more or less accurately. To encompass these views, I have defined activation as the information-driven reorganization of energy flows within and among populations of neuroglial units, leading to an overall increase in energy utilization in these populations [18]. Energy flows are coherent spatial and temporal changes in the energy turnover of neuroglial units that accompany information processing.

This definition includes metabolic energy turnover and energy turnover related to biophysical processes, such as the energy of electromagnetic fields. Energy flows propagate in specific directions along cellular structures (e.g., axons, dendrites, and synapses), but a constant level of metabolic energy turnover is required to maintain the mechanisms of electromagnetic-field generation, such as the transmembrane potential. Metabolic energy is therefore linked to electromagnetic energy, and the processing of incoming stimuli requires a spatial and temporal reorganization of the system. At rest, the organization of this system reflects predictive coding, and the degree of reorganization caused by stimulation depends on how far the stimulus deviates from the predicted state.

**Figure 2.**
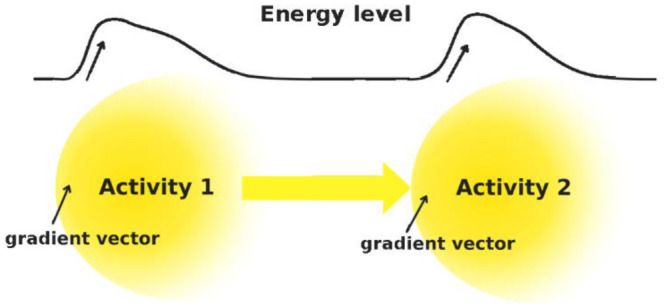
Gradients of energy in the direction of activity propagation. The arrival of nerve fibers at a neuroglial population leads to a synchronized burst of action potentials. This influx of neural signals produces a marked increase in energy consumption and utilization, driven by the high metabolic demands of neurotransmission and ion channel activity in the targeted area. The directions of these energy flows establish spatial coding at rest (causing activity differences between voxels) and change in response to sensory input [19].

Where fibers arrive at a neuroglial population, a burst of spikes corresponds to a marked increase in local energy consumption and utilization (Figure 2).

A substantial spatial change in energy between two points constitutes, in mathematical and physical terms, an energy gradient between them. The brain alters its activity not merely by raising activity in a single voxel, but by producing a specific pattern of increases and decreases across many voxels. The direction of this change (reflecting the flow from one voxel to another) contributes substantially to the spatial code (Figure 2). A steep gradient indicates a location of activity change in space, between two adjacent voxels. We have shown that gradients of brain activity occur in individual fMRI contrasts, are significant at the group level, and are specific to the type of stimulation [20]. The sources of these gradient vectors are also significant at the group level and are localized in the auditory cortex for auditory stimulation and in the visual cortex for visual stimulation.

Incoming stimulation can be represented as a field that rotates and rescales the gradient vectors of brain activity relative to their baseline directions and magnitudes [19]. This yields a computational approach for estimating the information-driven reorganization of energy flows within and among neuroglial populations [18]. The reorganization can be represented mathematically as transformation matrices or, more precisely, tensors.

Rather than observing only the outcome of stimulation, one can therefore study the dynamic interaction among the energy flows of the initial brain state and the transforming stimulation field. They provide a more detailed description of brain activation as defined above.

Gradients and sources in BOLD activity have shown substantial spatial overlap with those obtained from 3D distributed source reconstruction of EEG and MEG signals for the same stimulation [21]. This link to direct electromagnetic measures indicates that the energy gradients observed in fMRI are not merely an epiphenomenon but reflect the fundamental workings of neural circuits.

Assigning a spatial gradient vector to each voxel means that gradients describe the spatial structure (the activity differences between adjacent voxels) within a brain region. Activity gradients and the underlying energy flows may spatially encode the presented information. Spatial coding in fMRI has been validated by numerous decoding studies using multivariate pattern analysis (MVPA) [22],[23]. Moreover, for a given stimulus, brain areas that share information show similar spatial structure [24]. The goal of the brain may be to integrate information from spatial coding into a coherent image or sensation within an introspective virtual space [25]–[27].

Several caveats apply. The hemodynamic responses measured with fMRI and PET are only indirect proxies of neural activity. Furthermore, whole-brain metabolic energy consumption remains about 20% of total metabolic expenditure regardless of task demands [28],[29]. Within our framework, attention redistributes this fixed metabolic pool toward the regions generating the most informative internal representations. Increased perceptual load raises cellular metabolism in attended regions while reducing it in regions processing unattended stimuli, as shown by direct measurements of mitochondrial oxidative metabolism using broadband near-infrared spectroscopy [30]. Herculano-Houzel and Rothman [29] proposed a supply-limited framework in which cerebral blood flow operates as a closed system under physiological conditions. Thus, when I refer to energy increases in specific neural populations during stimulation, I mean a local reallocation within a globally constrained metabolic budget, not an increase in total brain energy supply.

It should also be noted that metabolic energy consumption is rate-dependent (energy turnover per unit time). Minimal energy required for equivalent information processing can vary with neural efficiency and circuit-level optimization. In addition, attention may fluctuate during highly demanding tasks, modulating the precision weighting of prediction errors and the associated metabolic demands.

Additionally, the correlation between fMRI-derived gradient patterns and EEG/MEG source reconstructions does not establish causation. Resting-state fMRI gradients may be influenced by non-neural factors, including regional variation in vascular density, vessel caliber, and vascular reactivity, which can introduce systematic spatial biases; EEG source reconstruction carries inherent spatial uncertainty owing to the ill-posed inverse problem. To establish a more direct causal link, researchers should employ laminar-resolved fMRI at ultra-high field strengths (7 T or higher) to distinguish cortical layers, electrocorticographic (ECoG) recordings in animal models, and simultaneous broadband NIRS–EEG recordings to measure cellular metabolic changes alongside electrophysiological signals.

## 5. Core postulates of the energy-embedded prediction framework

Building on the preceding discussion of predictive coding, neural energetics, and activity gradients, I can now state the core postulates of the Energy-Embedded Prediction (EEP) framework. The framework holds that internal predictive models are not merely abstract informational constructs but are physically instantiated and maintained by the brain's spatiotemporal energy dynamics. The following postulates set out its foundational principles.

*Postulate 1: Predictive states are energy landscapes*.

The brain's resting-state activity and its internal models of the environment are represented as spatially structured, stable energy landscapes, defined by spatial gradients of metabolic and electromagnetic free energy F across neural populations. The configuration of these gradients at rest constitutes the brain's baseline predictions about sensory input and environmental regularities.

To illustrate how a specific prediction maps onto the energy landscape, consider expecting a chair in an office. The brain's generative model encodes the statistical regularities of office environments, where chairs, desks, and windows are highly probable, whereas a caged monkey is not. These regular features are physically instantiated in the patterns of synaptic weights and connectivity shaped by experience. At rest, this pattern defines a stable energy landscape: A specific spatial configuration of free-energy gradients across neural populations that constitutes the baseline prediction. When a chair in the office is perceived, the sensory input closely matches the prediction; the prediction error is small, and the energy landscape is minimally perturbed. The spatial free energy gradients ∇F remain near their resting configuration. If a monkey in the office is perceived, a large prediction error arises because the input strongly violates the model's expectations. This error acts as a force that reshapes the local energy landscape: ∇F increase in magnitude and point in the directions that would most efficiently update the internal model to accommodate the unexpected input [1],[11]. The magnitude of ∇F thus reflects the size and precision-weighting of the prediction error. A highly unexpected event in a high-confidence context produces a high (steep) gradient, whereas a mildly unexpected event, or one in an uncertain context, produces a smaller (shallower) gradient. I propose that the increases in metabolic and electrical activity observed through neuroimaging in response to unexpected stimuli reflect this reshaping of the underlying free-energy landscape.

*Postulate 2: Prediction is energy-efficient conformity*.

Processing sensory input that matches an existing predictive model follows pre-established, energy efficient pathways facilitated by mechanisms such as long-term potentiation (LTP). This produces minimal reorganization of the underlying energy landscape, because neural activity conforms to the predicted state.

*Postulate 3: Prediction error drives energetic reorganization*.

A mismatch between incoming sensory information and the predictive model generates a prediction-error signal that acts as a thermodynamic force, driving a local or global reorganization of the brain's energy landscape. This reorganization is metabolically costly, requiring increased energy utilization to reshape activity gradients, recruit additional circuits, and establish new excitation loci.

*Postulate 4: Stimulation is a transformational field*.

External sensory stimulation can be formalized as a field (mathematically represented as a tensor, T) that operates on the brain's baseline energy-gradient field. This transformation rotates and scales the local gradient vectors, thereby altering the direction and magnitude of neural activity flows. The resulting pattern of brain activation is the observable outcome of the stimulation field-driven reorganization of energy flows.

*Postulate 5: Information is encoded in gradient structure*.

The information content of a stimulus, and its corresponding internal representation, is spatially encoded in the pattern of energy gradients; the spatial differences between neuroglial populations. The reorganization of these gradient patterns constitutes the brain's information processing.

*Postulate 6: The brain optimizes a free-energy gradient*.

The brain operates as a self-organizing, non-equilibrium system that seeks to minimize the surprisal (variational free energy) of its sensory inputs. In the EEP framework, this minimization is physically realized by the tendency of local thermodynamic forces to spontaneously flatten energy gradients (∇F → 0). Persistent, stimulus specific gradients, which I observe, reflect a dynamic balance in which these minimizing forces are counteracted by information encoding forces associated with ongoing prediction and error signaling.

**Figure 3.**
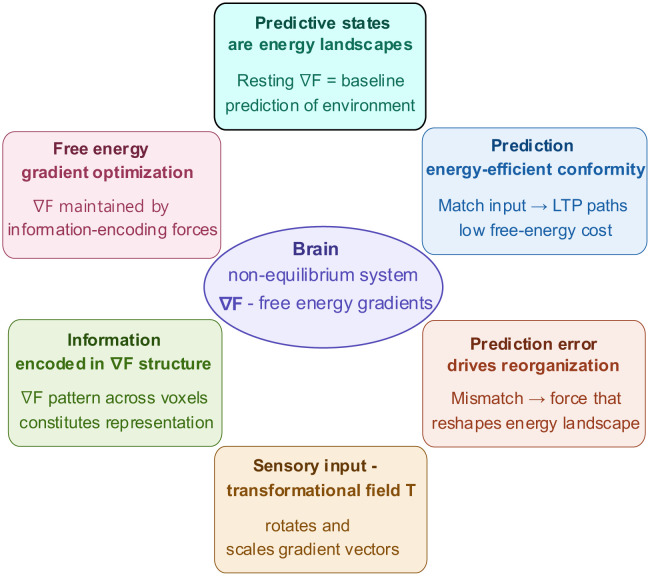
The Energy-Embedded Prediction (EEP) framework has six postulates. Postulate 1 states that the brain's resting-state free-energy gradient constitutes a baseline prediction of the environment; the predicted state is the energy landscape. Postulate 2 states that when sensory input matches this prediction, processing follows energy efficient, LTP-consolidated pathways at low metabolic cost. Postulate 3 states that when input violates the prediction, a thermodynamic force reshapes the local energy landscape in proportion to the precision-weighted prediction error. Postulate 4 states that external stimulation acts as a tensor transformation field T that rotates and rescales the gradient-vector field. Postulate 5 states that the resulting spatial pattern of gradients across voxels constitutes an information-bearing representation. Postulate 6 states that the system continuously optimizes the gradient field, balancing the thermodynamic drive toward equilibrium against the information-encoding forces that maintain structured, non-equilibrium states.

In summary, the EEP framework proposes that cognition emerges from a continuous, prediction-driven negotiation between information processing forces and the brain's fundamental tendency to settle into energy efficient states. Perception, learning, and adaptation are thus physical processes of energy landscape reconfiguration (Figure 3).

## 6. Gradients of activity and activity flows

The gradients of brain activity discussed above can be biophysically regarded as free-energy gradients and energy flows. This approach is conceptual and explanatory; modeling neural activity within the framework is a direction for future research. This section can be skipped by non-mathematical readers.

### 6.1. Gradients of energy and directions of activity propagation

In thermodynamics, the macroscopic state of a system generally depends on r extensive variables Xi (i = 1, ..., r). When supplemented by entropy S, the fundamental relation can be written in terms of internal energy as U = U [S, X₁, ..., Xr]. In many systems, the variables are volume V and particle number N, giving U = U [S, V, N]. Because entropy S is inconvenient to measure experimentally, the Legendre transformation is used to introduce the Helmholtz free energy F = F [T, V, N], where T is absolute temperature. Internal energy and free energy are thermodynamic potentials, whose meaning are considered further below.

If F depends on time t, the free-energy minimization principle [1],[11] requires its first time-derivative to tend to zero and its second to be positive. In Cartesian coordinates, the first derivative can be expressed as:

$$\frac{dF}{dt}=\frac{\partial F}{\partial x}\frac{dx}{dt}+\frac{\partial F}{\partial y}\frac{dy}{dt}+\frac{\partial F}{\partial z}\frac{dz}{dt}=\nabla F\cdot v=0,$$ (1)

where the free-energy gradient ∇F generally points in the same direction as its flow velocity v, and their dot product becomes zero because of the gradient-destroying nature of self-organizing, non-equilibrium living systems [11]. It follows that gradient of F in these voxels tends to zero:

$$\nabla F=\hat{x}\frac{\partial F}{\partial x}+\hat{y}\frac{\partial F}{\partial y}+\hat{z}\frac{\partial F}{\partial z}=0$$ (2)

However, our analysis demonstrates the existence of stimulation-specific gradients that are stable in time [20],[21]. The minimization principle is therefore counterbalanced by information-related processes and their associated forces.

In physics, the potential energy U(r) corresponding to a force F(r) can be expressed as an integral of F(r), where r is a vector pointing from the origin to a given location. The work W done by F(r) over a small displacement from r to r + dr is:

$$W\left(r\to r+dr\right)=F\cdot dr=F_{x}dx+F_{y}dy+F_{z}dz$$ (3)

The work of the external force is positive when the force acts in the direction of increasing energy (e.g., climbing a hill) and thus equals the change in potential energy:

W(r→r+dr)=dU=U(r+dr)−U(r) (4)

$$dU=\frac{\partial U}{\partial x}dx+\frac{\partial U}{\partial y}dy+\frac{\partial U}{\partial z}dz$$ (5)

The same work can also be expressed as a sum of force components along each axis:

$$W\left(r\to r+dr\right)=F\cdot dr=F_{x}dx+F_{y}dy+F_{z}dz$$ (6)

or as a function of the potential field against which the force acts:

$$W\left(r\to r+dr\right)=dU=\frac{\partial U}{\partial x}dx+\frac{\partial U}{\partial y}dy+\frac{\partial U}{\partial z}dz$$ (7)

Thus, the external force equals the gradient of U:

$$F=\hat{x}\frac{\partial U}{\partial x}+\hat{y}\frac{\partial U}{\partial y}+\hat{z}\frac{\partial U}{\partial z}=\nabla U$$ (8)

By analogy, the gradient of the free-energy field is written as follows (the field is denoted E here to avoid confusion with F used for force):

$$\nabla E=\hat{x}\frac{\partial E}{\partial x}+\hat{y}\frac{\partial E}{\partial y}+\hat{z}\frac{\partial E}{\partial z}$$ (9)

The value of this gradient represents an internal “force” that acts “downhill” along the free-energy values. This is not a conventional force but a generalized one, known as a thermodynamic force. As noted above, it typically drives everything within a voxel toward minimum free energy, yielding a zero gradient. A steady gradient therefore indicates additional forces that align with the field but counteract the thermodynamic force. These information encoding forces are associated with neural signal transmission, and their direction is defined by the equation for ∇F. Their exact nature may be complex and may relate to mechanisms that prevent neural activity from retracing the same path.

The thermodynamic driving force corresponds to the tendency of neural systems toward maximum entropy (thermodynamic equilibrium), expressed neurophysiologically as the degradation of organized neural activity toward baseline noise through metabolic heat dissipation and the dissipation of ion gradients across neuronal membranes. The information-encoding forces may correspond to the active maintenance of structured, non-equilibrium neural states, supported by: (a) Persistent sodium and calcium currents that sustain depolarization; (b) recurrent excitatory connectivity; (c) NMDA-receptor-mediated currents providing slow, voltage-dependent conductances; and (d) neuromodulatory systems, such as noradrenergic signaling, that regulate the signal-to-noise ratio and the precision of prediction-error signals. Because these two forces rest on distinct biophysical substrates, they can be manipulated independently. For example, pharmacological blockade of NMDA receptors should weaken the information force by disrupting sustained neural states, thereby reducing the amplitude of the measured free-energy gradient ∇F, whereas enhancement of noradrenergic signaling should strengthen the information encoding forces.

Equation (9) employs a scalar-field formulation to represent the spatial distribution of free-energy density across the brain's cortical tissue. A scalar field is appropriate here because the quantity of interest at each point is a single real number (the local free-energy value) rather than a vector or tensor. This continuous-field approximation assumes spatial continuity of the free-energy distribution and is valid when the spatial resolution of neuroimaging data (typically the voxel scale) is sufficiently coarse relative to the discrete cellular architecture that the aggregate energy state can be treated as a smooth function of spatial coordinates.

Using these relations, 3D images from fMRI and PET can be spatially differentiated to derive the gradient vector, or its projections, for each voxel. In the research discussed above, we used numerical gradients computed by finite-difference approximation, as implemented in the MATLAB gradient function. For the x dimension, FX = gradient (F, x):

$$F_{X}\left(i\right)=\frac{F\left(i+1\right)-F\left(i-1\right)}{x\left(i+1\right)-x\left(i-1\right)}$$ (10)

The first and last points in a row are first-order approximations (one a forward difference and the other a backward difference), while the intermediate points are second-order approximations. By established neuroimaging conventions for axis directions, positive projections along the X-axis indicate that the most significant activity changes within a cluster occur in the left–right direction.

### 6.2. Changes of energy flows due to stimulation

To obtain the diagonal matrix that serves as the tensor in this context, the gradient projections along each axis under one condition are divided by the corresponding projections under another condition. The values reported in neuroimaging [19] correspond to the t-values in the diagonal matrix of the following expression:

$$\left[\begin{array}{c} \nabla_{x}a_{1}\\ \nabla_{y}a_{1}\\ \nabla_{z}a_{1} \end{array}\right]=\left[\begin{array}{ccc} t_{11} & 0 & 0\\ 0 & t_{22} & 0\\ 0 & 0 & t_{33} \end{array}\right]\left[\begin{array}{c} \nabla_{x}a_{2}\\ \nabla_{y}a_{2}\\ \nabla_{z}a_{2} \end{array}\right]$$ (11)

Here, a₁ and a₂ denote the average activity values for conditions 1 and 2, respectively. To assess the strength of the transformation, the length of the diagonal vector [t₁₁, t₂₂, t₃₃] in the transformation matrix is calculated for each voxel. The group-level significance of this value is evaluated with a one-sample t-test in SPM and its non-parametric permutation equivalent in SnPM, with family-wise error (FWE) correction for multiple comparisons.

There is a distinction between the concept of activation used in this framework and its conventional usage in neuroimaging analysis. In standard analysis, “activation” is defined operationally through statistical thresholding procedures grounded in random field theory (RFT), which provides the mathematical framework for controlling family-wise error rates when making inferences about spatially extended statistical maps; the applied threshold determines, in binary fashion, which regions are declared active. In our framework, by contrast, activation is treated as a continuous energetic quantity; the degree of energy transformation and reorganization. Equation (11) describes a continuous tensor transformation that characterizes how the gradient field is reshaped by stimulation, and RFT-based thresholding is then applied to assess the significance of this continuous transformation at the group level.

To move beyond post-hoc description, I propose the following testable quantitative predictions. First, the norm of the t-vector (∥t∥ = √(t₁₁² + t₂₂² + t₃₃²)) should increase monotonically with the magnitude of the precision-weighted prediction error. In parametric oddball paradigms with graded deviance, I predict a positive relationship between deviance magnitude and ∥t∥, especially in the sensory cortex. Second, the relative magnitudes of the diagonal elements should reflect the dominant direction of information flow: In lower sensory cortices, the component aligned with the cortical hierarchy should dominate, whereas in higher-order association areas, the pattern should reverse. Third, directing attention toward a stimulus should increase ∥t∥ in the corresponding sensory representation, while directing attention away should decrease it, which is consistent with the role of attention in modulating the precision of prediction errors.

### 6.3. Relationship between the EEP framework and the Free Energy Principle

It is useful to position the EEP framework (Figure 3) relative to theoretical accounts, particularly the Free Energy Principle (FEP). as articulated by Friston [1],[11],[31] and the foundational work on resting-state brain energy metabolism by Raichle et al. [32].

Raichle et al. [32] established that the brain maintains a characteristic oxygen extraction fraction (OEF) at rest, defining a metabolically active default baseline. Within our framework, this default mode corresponds to a state of minimal predictive free-energy gradient, from which task related departures create the spatial energy gradients that, I propose, encode prediction and prediction error. The EEP framework thus aims to bridge the gap between the abstract information-theoretic quantities of the FEP and the physical energetic processes thought to sustain neural computation.

Although thermodynamic free energy cannot be measured directly in a living brain, I suggest that neuroimaging signals (fMRI, PET, and EEG) serve as indirect indicators of the underlying free-energy dynamics. In this sense, EEP addresses the physical energetic substrate of the computational processes described by the FEP. Indirect evidence supports these concepts; for example, Bruckmaier et al. [30] demonstrated that cellular metabolism in the visual cortex increases with perceptual load.

## 7. Conclusions

In conclusion, the energy flows between voxels represented by the spatial gradient vectors give rise to specific spatial structures within brain regions. At rest, these structures encapsulate internal representations; during sensory stimulation, energy flows reorganize, requiring increased energy input. Sensory stimulation can be represented as a transformation field that acts on the gradients of brain activity in the initial state, adjusting activity in response to sensory input.

By incorporating EEPs, this framework clarifies how activity gradients and their underlying flows support the spatial encoding of information. The integration of these spatial codes may play a crucial role in constructing a coherent image or sensation within an introspective virtual space, advancing our understanding of how the brain processes and interprets information. This perspective highlights the dynamic interplay between energy flows and cognitive functions.

The EEP framework described here is related to, but conceptually distinct from, FEP formulated by Friston [1],[11]. Both posit that the brain operates as a prediction machine that minimizes discrepancies between expected and observed states. The FEP employs variational free energy, a quantity rooted in Bayesian inference and information theory, to describe how the brain minimizes surprise about sensory inputs. Although Friston has shown that variational free energy can be formally analogous to thermodynamic free energy under certain strict assumptions, the two concepts originate in different domains. Thermodynamic free energy (Helmholtz free energy, F = U − TS) is a physical quantity describing the work-available energy of a system. The present framework proposes that the increases in metabolic and electrical activity observed through neuroimaging reflect increases in thermodynamic free-energy utilization while drawing on the FEP's insight that the brain acts as a Bayesian inference engine that generates and refines hypotheses to minimize prediction error. Isomura et al. [33] provided experimental validation of the FEP using in vitro networks of rat cortical neurons, showing that neurons self-organize to perform causal inference consistent with variational Bayesian inference, with changes in effective synaptic connectivity reducing variational free energy and connection strengths encoding parameters of the generative model. These findings provide empirical support for a neural substrate of free-energy minimization and strengthen the theoretical foundations of the present framework.

## 8. Perspectives

Brain predictions embedded in energy flows can illuminate the mechanisms underlying cognitive processes-attention, decision-making, language processing etc. The neuro-energetic signatures of predictive energy flows and their reorganization can be detected using neuroimaging techniques e.g., functional magnetic resonance imaging, positron emission tomography, and electroencephalography. Since updating predictive models is closely related to learning and memory formation, neuro-energetic principles can also provide insight into how the brain learns and stores information.

Moreover, understanding the energetic aspects of brain predictions can inform the development of more biologically inspired artificial neural networks and machine-learning algorithms. Applying energy-efficient coding principles to artificial systems may enable them to make more accurate predictions. The same principles can help explain how the brain processes and integrates sensory information across modalities (e.g., vision, audition, and touch).

From a medical perspective, abnormalities in predictive internal models have been associated with neurological and psychiatric disorders, including schizophrenia, autism, and Parkinson's disease. By studying the neuro-energetic correlates of prediction-error models in these conditions, we may develop new diagnostic tools and intervention strategies.

## Glossary of key terms

**∇F (free-energy gradient):** The spatial gradient of Helmholtz free energy across brain tissue, computed as the vector of partial derivatives of F with respect to spatial coordinates. It is operationalized at the voxel scale as the numerical gradient of the activity field estimated from neuroimaging data.

**Energy (activity) flow [18]:** Coherent spatial and temporal changes in the energy turnover of neuroglial units that accompany information processing; that is, the directed, energy-consuming transfer of metabolic energy between spatially adjacent neural populations, which creates free-energy gradients.

**Information-encoding forces:** Generalized forces that maintain non-equilibrium, structured neural states against thermodynamic dissipation.

**Transformation tensor (T):** A second-order tensor characterizing the local strength and directionality of the transformation between predicted and observed energy states at each voxel. Its diagonal elements (t₁₁, t₂₂, t₃₃) represent the transformation magnitude along each spatial axis.

**Spatial coding:** The representation of information through the spatial distribution of free-energy gradients across cortical tissue, as opposed to temporal coding (spike timing) or rate coding (firing rate).

## Use of AI tools declaration

The author declares they have not used Artificial Intelligence (AI) tools for the creation of this article.
